# Regulation of Osteoclast Growth and Fusion by mTOR/raptor and mTOR/rictor/Akt

**DOI:** 10.3389/fcell.2017.00054

**Published:** 2017-05-18

**Authors:** Kerstin Tiedemann, Damien Le Nihouannen, Jenna E. Fong, Osama Hussein, Jake E. Barralet, Svetlana V. Komarova

**Affiliations:** ^1^Faculty of Dentistry, McGill UniversityMontreal, QC, Canada; ^2^Shriners Hospital for Children-CanadaMontreal, QC, Canada; ^3^Department of Surgery, Faculty of Medicine, McGill UniversityMontreal, QC, Canada

**Keywords:** osteoclast, monocyte fusion, cell growth, mTOR, Akt

## Abstract

Osteoclasts are giant bone cells formed by fusion from monocytes and uniquely capable of a complete destruction of mineralized tissues. Previously, we have demonstrated that in energy-rich environment not only osteoclast fusion index (the number of nuclei each osteoclast contains), but also cytoplasm volume per single nucleus was increased. The goal of this study was to investigate the regulation of metabolic sensor mTOR during osteoclast differentiation in energy-rich environment simulated by addition of pyruvate. We have found that in the presence of pyruvate, the proportion of mTOR associated with raptor increased, while mTOR-rictor-mediated Akt phosphorylation decreased. Inhibition of mTOR with rapamycin (10 nM) significantly interfered with all aspects of osteoclastogenesis. However, rapamycin at 1 nM, which preferentially targets mTOR-raptor complex, was only effective in control cultures, while in the presence of pyruvate osteoclast fusion index was successfully increased. Inhibition of Akt drastically reduced osteoclast fusion, however in energy-rich environment, osteoclasts of comparable size were formed through increased cytoplasm growth. These data suggest that mTOR-rictor mediated Akt signaling regulates osteoclast fusion, while mTOR-raptor regulation of protein translation contributes to fusion-independent cytoplasm growth. We demonstrate that depending on the bioenergetics microenvironment osteoclastogenesis can adjust to occur through preferential multinucleation or through cell growth, implying that attaining large cell size is part of the osteoclast differentiation program.

## Introduction

Osteoclasts are bone cells uniquely capable of a complete destruction of mineralized tissues. Osteoclast function is required physiologically during development for proper bone shaping and tooth eruption, as well as in adult life to provide access to bone-stored minerals (Feng and Teitelbaum, 2013; Segeletz and Hoflack, 2016). Pathologically, osteoclasts are responsible for bone destruction in degenerative, inflammatory and metabolic bone disorders (Henriksen et al., 2011; Boyce, 2013). Osteoclasts are known as giant cells formed by fusion of monocytes, cells of hematopoietic origin. Single osteoclasts can contain between 3 and 100 nuclei, varying in diameter between 10 and 300 μM (Gardner, 2007; Akchurin et al., 2008; Kopesky et al., 2014). Importantly, it has been demonstrated that large osteoclasts are more likely to be observed during pathological bone resorption, and are more active and more responsive to environmental stimuli (Lees and Heersche, 1999, 2000; Lees et al., 2001; Trebec et al., 2007). A number of molecular mediators were reported to be important for osteoclast fusion, including DC-STAMP (Yagi et al., 2005), CD9 (Ishii et al., 2006), V0 subunit d2 of the lysosomal H^+^-ATPase (ATP6V0d2) (Kim et al., 2006), and Fra-2-regulated LIF/LIF-receptor signaling (Bozec et al., 2008). Nevertheless, the complete sequence of events leading from stimulation of osteoclastogenesis by receptor activator of nuclear factor κB ligand (RANKL) to formation of large polykarions capable of bone destruction is incompletely understood.

We have previously demonstrated that supplementation of cultures with moderate amounts of metabolic energy substrates, such as pyruvate, stimulates osteoclastogenesis resulting in formation of more and larger osteoclasts (Fong et al., 2013). We have found that addition of pyruvate augmented mitochondrial respiration, resulting in a three-fold increase in ATP levels and inhibition of AMP-activated protein kinase (AMPK). AMPK is a metabolic sensor stimulated by increase in AMP/ATP ratio that signifies cell inability to cope with the energy demands (Finley and Haigis, 2009). AMPK acts to decrease the cell metabolic expenditure and to improve energy production by inducing mitochondrial biogenesis and fatty acid oxidation. AMPK counterpart is mTOR, which is suppressed when nutrients are limited. mTOR exists in two complexes. mTORC1 (with raptor, PRAS40 and mLST8) regulates protein synthesis through phosphorylation of an activator of translation, ribosomal protein p70 S6 kinase (S6K) and an inhibitor of translation eukaryotic translation initiation factor 4E binding protein 1 (4E-BP1) (Foster and Toschi, 2009; Laplante and Sabatini, 2013). mTORC2 (with rictor, mSIN1, proctor, and mLST8) affects cytoskeletal organization and survival (Sarbassov et al., 2005a; Gaubitz et al., 2016). mTOR signaling was shown to be important for osteoclast formation and survival (Glantschnig et al., 2003; Sugatani and Hruska, 2005; Hu et al., 2016; Dai et al., 2017), especially in the setting of experimental bone metastasis (Hussein et al., 2012; Abdelaziz et al., 2014, 2015; Mercatali et al., 2016). Akt has been reported as a target of mTORC2 complex (Sarbassov et al., 2005a), as well as an upstream regulator of mTOR activity as part of the PI3K/Akt pathway (Lee et al., 2007). Akt has also been implicated in regulation of osteoclast differentiation and survival (Gingery et al., 2003; Sugatani and Hruska, 2005; Kawamura et al., 2007; Hu et al., 2008; Kwak et al., 2008). The role of mTOR in cell growth in general is well appreciated, however, its specific contribution to regulation of osteoclast size is not completely understood.

The goal of the present study was to examine the role of mTOR signaling in regulating osteoclast size through fusion and/or growth. We used supplementation with low levels of pyruvate (1 mM) to simulate an energy-rich environment which we previously found to actively modulate osteoclast size.

## Materials and methods

### Cell culture reagents

Fetal bovine serum (FBS) was from HyClone (SH 30396-03), Dulbecco's modified Eagle's medium (DMEM, 319-020-CL), pyruvate (600-110-EL), L-glutamine (609-065-EL), penicillin/streptomycin (450-201-EL), trypsin/ethylenediamine tetraacetic acid (T/E, 325-042-EL) were from Wisent Inc. Rapamycin (PHZ1233) was from Sigma-Aldrich Co. Akt inhibitor (1L6-Hydroxymethyl-chiro-inositol-2-(R)-2-O-methyl-3-O-octadecyl-sn-glycerocarbonate) was from Calbiochem (124005). Recombinant human M-CSF (300-25) was from Peprotech Inc. Recombinant glutathione S-transferase-soluble RANKL was purified from the clones kindly provided by Dr. M.F. Manolson (University of Toronto).

### Osteoclast cultures

This study was carried out in accordance with the recommendations of the Canadian Council on Animal Care. The protocol was approved by the McGill University Animal Care Committee. Mouse bone marrow cells were collected from 6 weeks old C57BL6/J mice (Charles River) as described previously (Boraschi-Diaz and Komarova, 2016). Cells were cultured in 75 cm^2^ tissue culture flasks (1.5 × 10^7^ cells per flask) with M-CSF (25 ng/ml) for 24 h, then non-adherent cells were collected and plated at 5 × 10^4^ cells/cm^2^ in α-MEM supplemented with 1% penicillin-streptomycin and 10% FBS, M-CSF (50 ng/ml) and RANKL (100 ng/ml). Medium was changed every other day. RAW 264.7 cells (ATCC) were cultured in 25 cm^2^ tissue culture flasks in DMEM supplemented with 1% penicillin-streptomycin and 10% FBS. To generate osteoclasts, RAW 264.7 cells were plated at 5 × 10^3^ cells/cm^2^. On day 1 and 3, medium was changed and M-CSF (50 ng/ml) and RANKL (50 ng/ml) were added. Cell cultures were fixed using 4% paraformaldehyde and stained for tartrate-resistant acid phosphatase (TRAP, Sigma-Aldrich Co, 387A). Osteoclasts were identified as multinucleated (more than 3 nuclei) TRAP-positive cells and were further characterized by image analysis using PixeLINK Capture SE® software (PixeLINK) and Image J. For each experimental condition, the cell surface area and nuclei number of 35–100 osteoclasts were evaluated.

### Confocal microscopy

Osteoclasts were generated from RAW 264.7 cells on glass coverslips. Fixed cells were incubated with the fluorescent lipophilic membrane probe DiI (5 μl/ml, Vybrant® DiI, Invitrogen™, V-22885), Alexa 488-conjugated Phalloidin (Invitrogen A12379), and DAPI stain (Invitrogen D1306), and visualized with confocal microscope (LSM510, Carl Zeiss Inc.) with Plan Apochromat 63×/1.4 NA oil objective. Image stacks were collected and 3D shape was reconstructed and analyzed using LSM510 software. Images of at least 20 fields/condition were used to evaluate osteoclast height.

### Protein extraction and immunoblotting

Cell lysates were extracted in RIPA lysis buffer containing 50 mM Tris, pH 7.4, 150 mM NaCl, 1% Nonidet P-40, 1 mM EDTA, 1 mg/ml aprotinin, 2 mg/ml leupeptin, 0.1 mM phenylmethylsulfonyl fluoride, 20 mM sodium fluoride, 0.5 mM sodium orthovanadate, and centrifuged at 12,000 g for 10 min at 4°C. Supernatant was collected, and protein was measured using a Quant-iT™ protein assay kit (Invitrogen). Twenty to forty micrograms of lysates was separated on a 7.5 or 12% SDS-PAGE and transferred to a nitrocellulose membrane (0.45 μm, 162-0115, Bio-Rad) using 10 mM sodium borate buffer. The membranes were blocked in 5% milk in TBST buffer (10 mM Tris-HCl, pH 7.5, 150 mM NaCl, 0.05% Tween 20) for 1 h at room temperature, and incubated overnight at 4°C with primary antibodies: p-Akt (1:1,000, #9271, Cell Signaling), Akt (1:1,000, #9272, Cell Signaling), p-4E-BP1 (1:1,000, #9451, Cell Signaling), 4E-BP1 (1:1,000, #9452, Cell Signaling), p-S6K (1:1,000, #9234, Cell Signaling), p-TSC2 (1:1,000, #3615p, Cell signaling), TSC2 (1:1,000, #4308p, Cell signaling), α-tubulin (1:5,000, T9026, Sigma), and calnexin (1:1,000, NBP2-43765, Novus Biologicals). The blots were visualized with horseradish peroxidase-conjugated secondary antibodies (anti-rabbit, 170–5046; anti-mouse, 170–5047 Bio-Rad) and a chemiluminescence system (Super signal West Pico; 34080, Pierce).

### Immunoprecipitation

Lysis buffer with 0.3% CHAPS instead of 1% triton was used to preserve the integrity of the mTOR complexes. Four micrograms of mTOR antibody (1:200, #2972, Cell Signaling) were added to the cleared cell lysates and incubated with rotation for 90 min. Twenty-five microliters of 50% slurry of protein G-sepharose were added and incubation continued for 1 h. Immunoprecipitates captured with protein G-sepharose were washed with the CHAPS lysis buffer, centrifuged briefly and the supernatant was removed from the protein G-Sepharose. Thirty microliters of loading buffer were added to the beads, boiled at 95–100°C for 5 min, loaded on an SDS-PAGE, and analyzed by immunoblotting for mTOR (1:200; #2972; Cell Signaling), raptor (1:200; #2280; Cell Signaling), and rictor (1:200; #2114; Cell Signaling).

### RNA isolation and RT-PCR

Total RNA was isolated from primary cultures using the RNeasy mini kit and QIAshredder columns (Qiagen, 74104 and 79654). For real-time PCR, 1 μg of total RNA was reverse transcribed using a cDNA archive kit (Applied Biosystems, 74322171). Real-time PCR was performed using 7,500 Applied Biosystems instrument using SYBR Green Universal PCR Master Mix (Applied Biosystems, 4367659) and the following primers: *Dcstamp* forward 5′-CTTCCGTGGGCCAGAAGTT-3′, and reverse 5′-AGGCCAGTGCTGACTAGGATGA-3′ and *Gapdh* forward, 5′-TTCCGTGTTCCTACCCCCAA-3′, and reverse, 5′-GATGCCTGCTTCACCACCTT-3′.

### Statistical analysis

Data are presented as representative images, representative experiments, or as means ± standard error of the mean, with n indicating the number of independent experiments. Differences were assessed by Student *t*-test or ANOVA with Tukey *post-hoc* test and accepted as statistically significant at *P* < 0.05.

## Results

### Larger osteoclasts are formed in the presence of pyruvate

**Figure 1:**
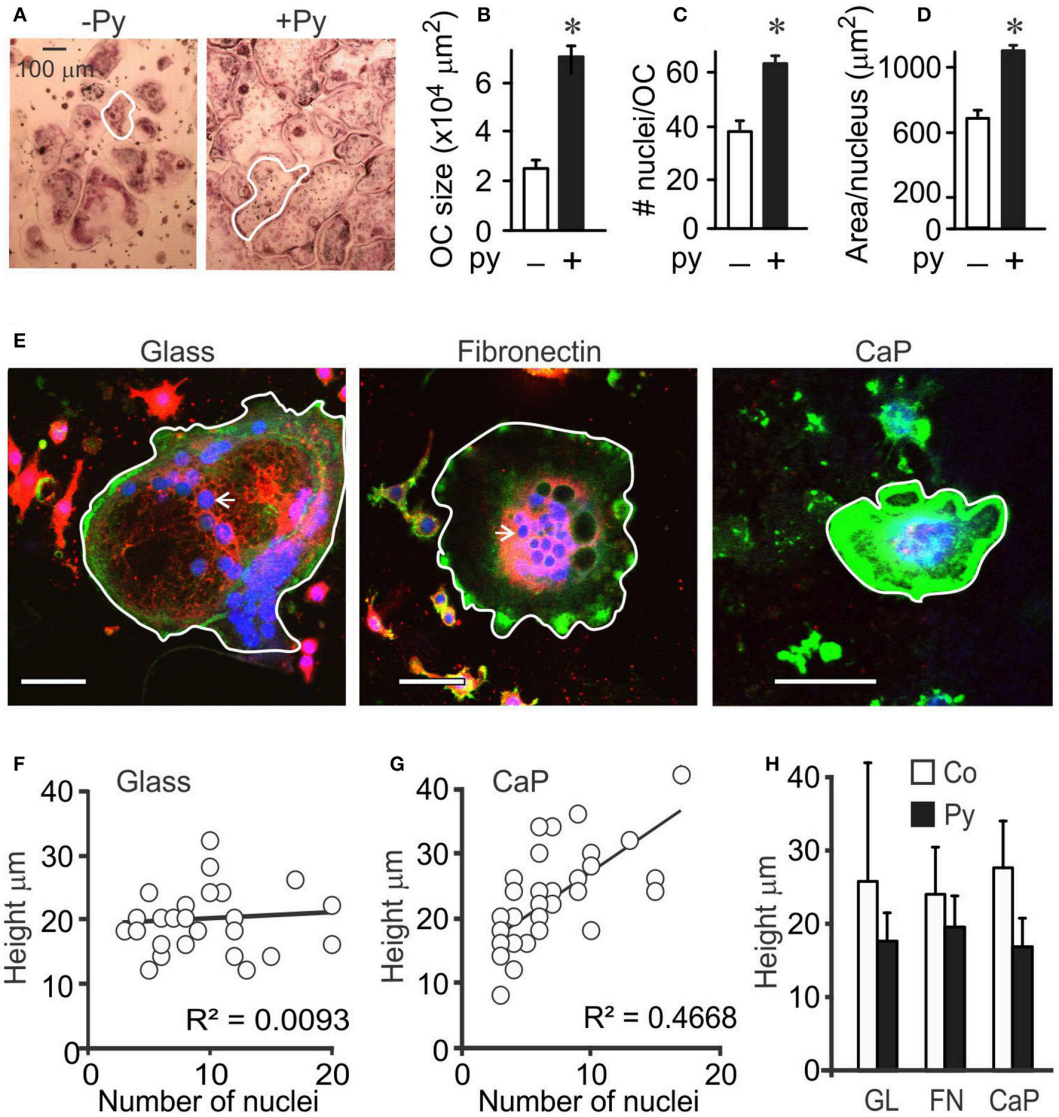
**Osteoclast size is increased in the presence of pyruvate**. Osteoclast precursors were treated with RANKL (50 ng/ml) for 4 days without (white bars) or with (black bars) pyruvate. **(A)** Representative images of osteoclasts generated from RAW 264.7 cells in the absence or presence of pyruvate (Py, 1 mM). Scale bar applies to both images, white outlines indicate representative osteoclast sizes. **(B–D**) Average osteoclast planar area **(B)**, number of nuclei per osteoclast **(C)**, and area per nucleus **(D)**. Data are means ± SE; *n* = 3 independent experiments. ^*^p < 0.05 indicates statistical significance assessed by paired *t*-test compared to samples cultured without pyruvate. **(E–H)** RAW264.7 cells were treated with RANKL (50 ng/mL) for 5 days, re-plated on glass coverslips uncoated (glass), coated with fibronectin (FN), or coated with calcium phosphate (CaP), cultured for 24 h without or with pyruvate, fixed and stained for actin using Alexa 488-conjugated phalloidin (green), membrane using DiI (red), and nuclei using DAPI (blue). **(E)** Representative images of osteoclasts on uncoated glass (left), fibronectin-coated glass (middle), and calcium-phosphate (right). Scale bar is 100 μm, white outlines indicate representative osteoclast sizes, white arrows point at single osteoclast nucleus. **(F,G)** The correlation between the number of nuclei and height of osteoclasts was assessed for 32–48 cells cultured on glass **(F)**, or calcium phosphate **(G)**. **(H)** Average osteoclast height in samples cultured on different substrates with or without pyruvate. Data are means ± SD, *n* = 14–31 osteoclasts per condition, no significant difference.

Previously, we have demonstrated that addition of moderate amounts of pyruvate stimulates osteoclast formation and growth (Fong et al., 2013). We further examined the effect of pyruvate (1 mM) on osteoclast size (Figure 1). Addition of pyruvate resulted in formation of larger osteoclasts (Figures 1A,B), both through an increase in fusion, evident by higher osteoclast nucleation (Figure 1C) and through an increase in cytosolic growth evident by increased planar area per nucleus (Figure 1D). Since osteoclasts are known to considerably change their shape (Holloway et al., 1997; Komarova et al., 2003), we examined if planar cell area accurately reflects osteoclast size. Using confocal analysis, we examined the height of osteoclasts containing different numbers of nuclei (Figure 1E). On non-resorbable substrates, such as glass (Figure 1F) or fibronectin (data not shown), osteoclasts containing different nuclei number exhibited similar heights, while on calcium phosphate significant correlation between the height and the number of nuclei in the osteoclast was observed (Figure 1G), likely reflecting resorption–associated change in osteoclast shape. Nevertheless, osteoclast height was similar in the absence or presence of pyruvate and independent of the differentiation substrate (glass, fibronectin, or calcium phosphate; Figure 1H), indicating that osteoclast planar area reflects osteoclast size.

### mTOR is regulated by pyruvate

**Figure 2:**
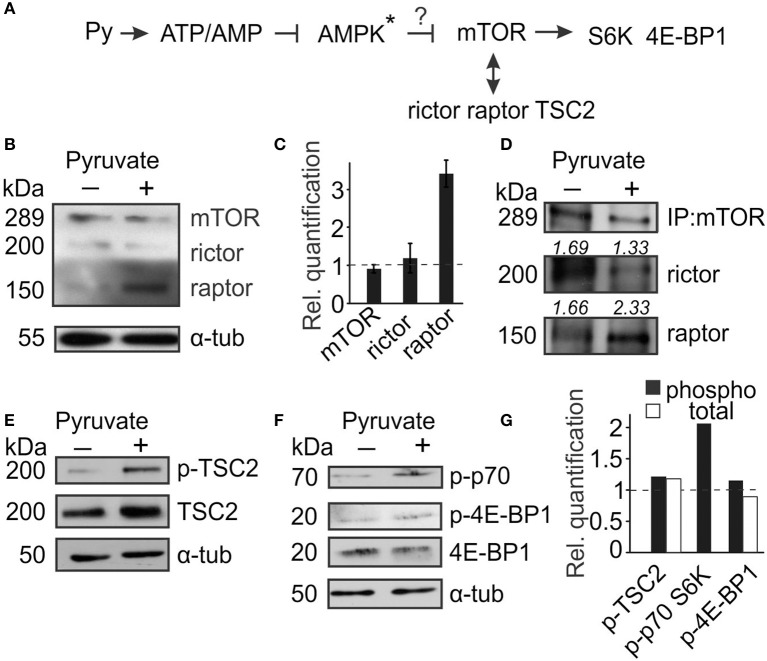
**mTOR signaling is altered in the presence of pyruvate**. Osteoclast precursors were treated for 6 h–2 days with RANKL, with or without pyruvate (1 mM) and cell lysates were collected. **(A)** Schematics of mTOR signaling pathway examined. Star ^*^ indicates that the role of AMPK was established in the previous study (Fong et al., 2013). **(B,C)** After 2 days of culture, the levels of mTOR, rictor and raptor were assessed. **(B)** Representative immunoblots, α-tubulin was used as a loading control. **(C)** Quantification of protein levels in cultures treated with pyruvate relative to untreated cultures (dashed line). **(D)** Cell lysates shown in **(B)** were immunoprecipited with anti-mTOR antibody and levels of raptor and rictor bound to mTOR were assessed. The number above the blot indicates rictor or raptor protein levels relative to mTOR. **(E–G)** Cell lysates were collected after 6–12 h, and the levels of phospho- and total TSC2 **(E)**; phospho- and total 4E-BP1, and phospho-p70S6K **(F)** were examined; α-tubulin was used as a loading control. **(G)** Immunoblots were quantified for levels of phosphorylated proteins to total proteins (black bars), except for p-p70S6K, which was normalized to tubulin; or levels of total proteins to tubulin (white bars) and expressed relative to cultures without pyruvate (dashed line).

We have previously demonstrated that the effects of pyruvate on osteoclastogenesis are mediated by AMPK (Fong et al., 2013). Since mTOR is a known downstream target of AMPK, we considered its role in regulation of osteoclast size (Figure 2A). mTOR can form complex with raptor, mTORC1, which regulates protein synthesis, or with rictor, mTORC2, which regulates cytoskeleton organization. We assessed the expression of mTOR, rictor, and raptor in control and pyruvate-treated cultures, and found that in cells treated with pyruvate, mTOR protein levels did not change, but expression of raptor strongly increased (Figures 2B,C). We next examined if addition of pyruvate can affect mTOR distribution between mTORC1 and mTORC2. Cell lysates were collected after 48 h treatment of RAW 264.7 cells with RANKL in the absence or presence of pyruvate, and immunoprecipitated with mTOR antibody. Treatment with pyruvate increased the amount of raptor co-precipitated with mTOR, while to a smaller degree decreasing the amount of rictor (Figure 2D), suggesting a shift toward preferential formation of mTORC1 in energy-rich conditions. Another potential regulator of mTOR signaling, TSC2, was also affected by addition of pyruvate (Figure 2E). To confirm mTORC1 activation in pyruvate-treated cultures, we assessed its direct phosphorylation targets p70S6K and 4E-BP1. Treatment with pyruvate for 6 h had minor but positive effects on phosphorylation of 4E-BP1 and strongly increased phosphorylation of p70S6K (Figures 2F,G).

### mTOR is important for osteoclastogenesis

**Figure 3:**
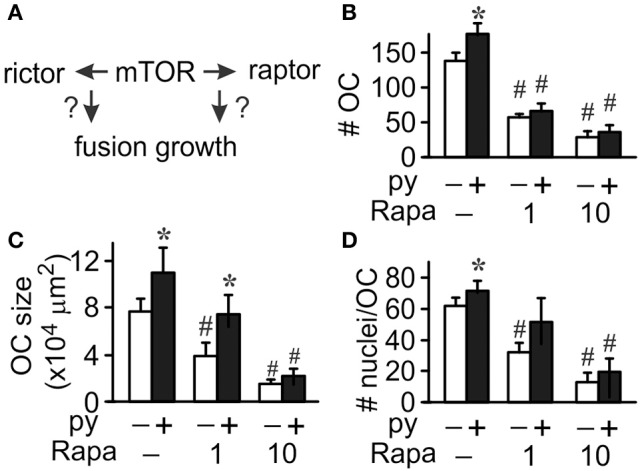
**mTOR pathway is important for osteoclastogenesis**. Osteoclast precursors were treated for 4 days with RANKL, without (white bars) or with (black bars) pyruvate (1 mM), and in the absence or presence of mTOR inhibitor rapamycin (Rapa, 1 or 10 nM) and the effect of mTOR inhibition on osteoclast fusion and growth was assessed **(A)**. **(B)** Average number of osteoclasts formed in different conditions. **(C)** Average osteoclast size. **(D)** Average number of nuclei per osteoclast. Data are means ± SEM, *n* = 5–7 independent experiments, ^*^p < 0.05 indicates statistical significance for the effects of pyruvate at the same levels of rapamycin; ^#^*p* < 0.05 indicates statistical significance for the effects of rapamycin at the same levels of pyruvate assessed by paired *t*-test.

We next used rapamycin to assess the role of mTOR in osteoclast differentiation (Figure 3A). At low concentration (l nM) rapamycin was shown to target raptor of the mTORC1 complex, while at high concentration (more than 10 nM) it inhibits both mTORC1 and mTORC2 complexes (Acosta-Jaquez et al., 2009). We found that in control cultures addition of 1 or 10 nM rapamycin results in significant, dose-dependent reductions in osteoclast numbers, size, and nucleation (Figure 3, white bars). However, the presence of pyruvate modified the effectiveness of rapamycin at low (l nM), but not high (10 nM) concentration. While 1 nM rapamycin still significantly inhibited osteoclast formation in pyruvate-supplemented cultures (Figure 3B), it was unable to reduce the size and nucleation of osteoclasts that were formed (Figures 3C,D). Thus, mTOR activity is required in both normal and high-energy environment, however only in high energy environment osteoclast size can be successfully increased in the absence of mTOR/raptor complex, likely through increased fusion mediated by mTOR/rictor complex.

### Akt phosphorylation is regulated by pyruvate

**Figure 4:**
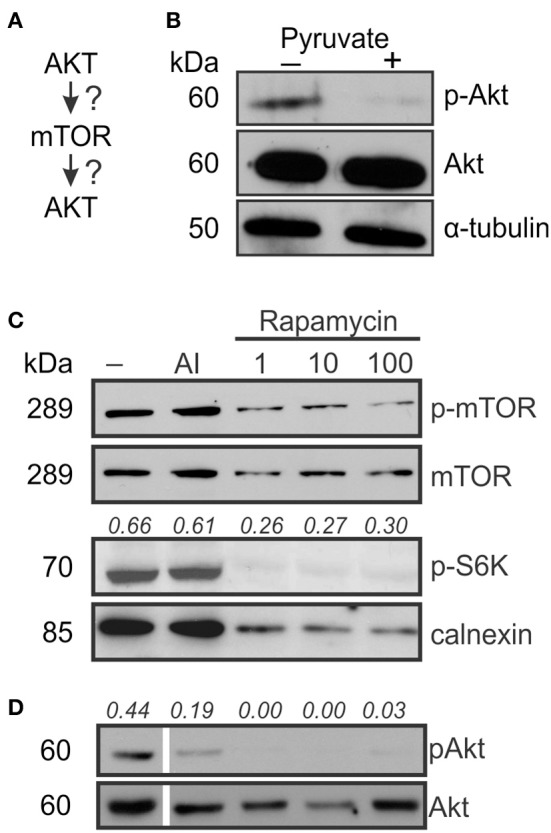
**AKT acts down-stream of mTOR signaling**. To examine if Akt is upstream or downstream of mTOR signaling **(A)**, osteoclast precursors were treated with RANKL (50 ng/ml) for 2–3 days with and without pyruvate (1 mM). **(B)** The levels of phosphorylated (Ser473) and total Akt in control and pyruvate-supplemented cultures, α-tubulin was used as a control for protein loading. **(C)** Cells cultured without pyruvate were exposed to Akt inhibitor (AI, 5 μM) or rapamycin (1, 10, or 100 nM), and mTOR phosphorylation, total mTOR levels and phosphorylation of p70S6K was assessed, calnexin was used as a protein loading control. The numbers above the blots indicate phospho-S6K levels relative to calnexin. **(D)** The effect of Akt inhibitor or rapamycin on Akt phosphorylation. The numbers above the blots indicate phospho-Akt levels relative to total Akt. To be noted, all the lanes were parts of the same gel, however, the lane order was changed, resulting in discontinuity.

Osteoclast differentiation involves multiple pathways including Akt signaling (Gingery et al., 2003), which in turn was shown to act both upstream and downstream of mTOR signaling (Sarbassov et al., 2005b; Ma and Blenis, 2009; Figure 4A). We examined if pyruvate affects Akt during osteoclastogenesis and found that Akt phosphorylation was suppressed in the presence of pyruvate (Figure 4B). To examine if Akt acts upstream or downstream of mTOR, we examined phosphorylation of both targets as well as mTOR-regulated p70S6K in cells cultured without pyruvate in the presence of specific inhibitors of Akt and mTOR. As expected, addition of rapamycin decreased mTOR and p70S6K phosphorylation (Figure 4C). In addition, Akt phosphorylation was dramatically decreased in rapamycin-treated cells (Figure 4D). In contrast, inhibition of Akt, while predictably reducing Akt phosphorylation, did not affect phosphorylation of mTOR or p70S6K (Figures 4C,D). Taken together, these data indicate that during osteoclast differentiation, Akt acts downstream of mTOR, and that exposure to pyruvate results in downregulation of Akt phosphorylation.

### Akt pathway mediates osteoclast fusion

**Figure 5:**
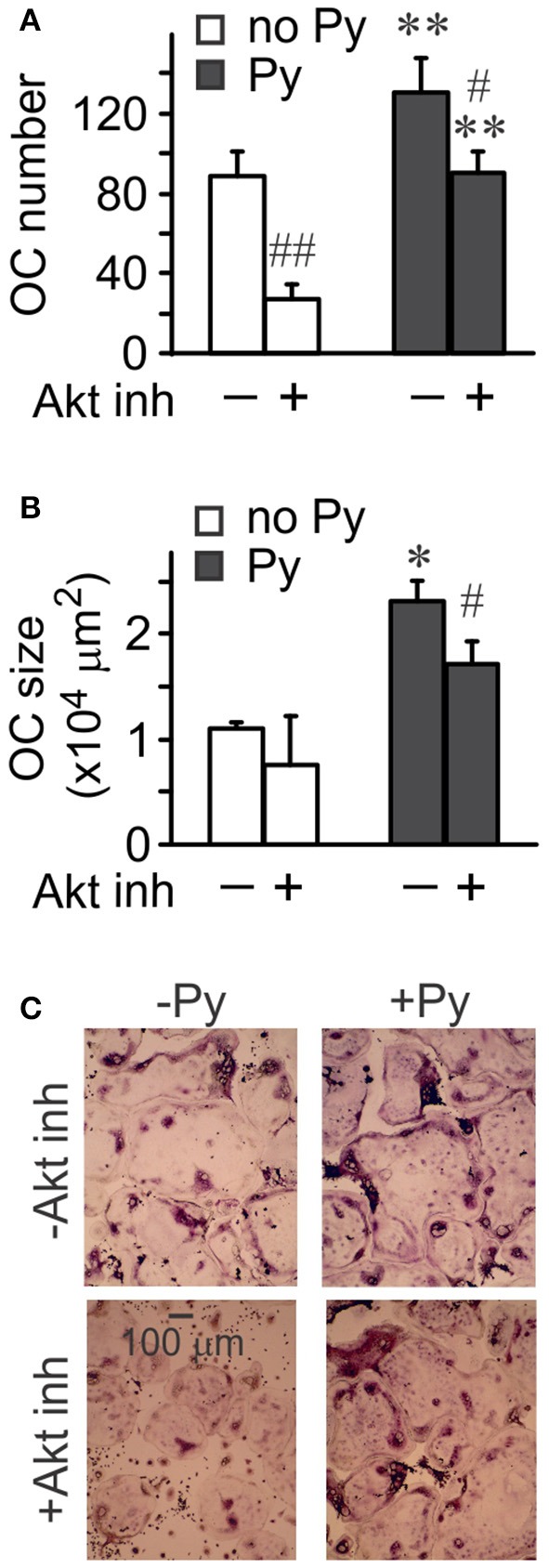
**Akt signaling is important for osteoclastogenesis**. Osteoclast precursors were treated for 4 days with RANKL (50 ng/ml), without or with pyruvate (1 mM), and in the absence or presence of Akt inhibitor (5 μM). **(A)** Average numbers of osteoclasts formed in different conditions. **(B)** Average osteoclast size. **(C)** Representative images of osteoclasts generated in the absence or presence of pyruvate and Akt inhibitor (5 μM). Scale bar applies to all images. Data are means ± SE, *n* = 3 independent experiments, ^*^p < 0.05, ^**^p < 0.01 indicate statistical significance for the effects of pyruvate at the same levels of Akt inhibitor; ^#^*p* < 0.05, ^##^*p* < 0.01 indicate statistical significance for the effects of Akt inhibitor at the same levels of pyruvate assessed by paired *t*-test.

**Figure 6:**
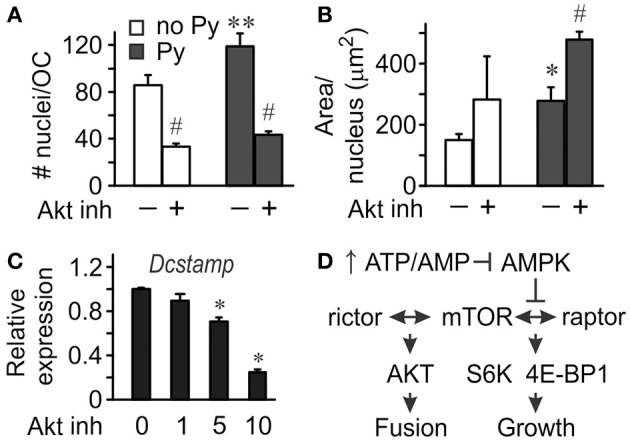
**Akt signaling mediates osteoclast fusion**. Osteoclast precursors were treated for 4 days with RANKL, without or with pyruvate (1 mM), and in the absence or presence of Akt inhibitor (5 μM). **(A)** Average numbers of nuclei per osteoclast. **(B)** Average planar area per nucleus. Data are means ± SE, *n* = 3 independent experiments, ^*^p < 0.05, ^**^p < 0.01 indicates statistical significance for the effects of pyruvate at the same levels of Akt inhibitor; ^#^*p* < 0.05 indicates statistical significance for the effects of Akt inhibitor at the same levels of pyruvate assessed by paired *t*-test. **(C)** Relative expression of *Dcstamp* in osteoclast cultures treated with pyruvate and Akt inhibitor at 1, 5, 10 μM. Data are means ± SD, *n* = 3 replicates, ^*^p < 0.05 indicates statistical significance for the effects of Akt inhibitor assessed by ANOVA. **(D)** Schematics of proposed signaling events mediating the effect of bioenergetics on osteoclast fusion and growth.

To investigate the role of Akt in osteoclastogenesis, we used an Akt inhibitor. Significantly fewer osteoclasts were formed in the presence of Akt inhibitor both in the absence and presence of pyruvate (Figure 5A), even though the effect of Akt inhibition was less pronounced in the presence of pyruvate, and pyruvate significantly increased osteoclastogenesis when Akt was inhibited (Figure 5A). In contrast, inhibition of Akt was not as effective in reducing the size of osteoclasts that were formed (Figures 5B,C). We next examined if osteoclast size increased through cell fusion or through cytoplasm growth. Average number of nuclei per single osteoclast was dramatically reduced when Akt was inhibited both in the absence and presence of pyruvate, consistent with inhibition of precursor fusion (Figure 6A). However, osteoclast surface area per nucleus was increased in the presence of Akt inhibitor, especially when cells were supplemented with pyruvate (Figure 6B), indicating that osteoclasts increase in size through fusion-independent cell growth, and that this process requires an energy-rich environment. In keeping with the role of Akt in osteoclast fusion, we have found that Akt inhibition dose-dependently reduced the gene expression of the key osteoclast fusion factor, DC-STAMP (Figure 6C).

## Discussion

Taken together our data indicate that two distinct processes occur simultaneously during the formation of large osteoclasts: continuous fusion and fusion-independent cytoplasm growth. Regulation by mTOR appears to be critical in defining the relative contribution of these processes, with mTOR-raptor complex, which is known to control protein synthesis, being responsible for fusion-independent growth; and mTOR-rictor mediated Akt signaling stimulating osteoclast fusion (Figure 6D). This regulation is flexible and responsive to changes in cell microenvironment. In an energy-rich environment the proportion of mTOR associated with raptor increases, while mTOR-rictor-mediated Akt phosphorylation decreases, resulting in increase in fusion-independent cytoplasm growth. Importantly, in energy-rich environment, osteoclasts of comparable size are formed even when fusion is drastically reduced by Akt inhibition, suggesting that cytoplasm growth can compensate for reduced fusion. These data further imply that increasing cell size is an important part of osteoclastogenesis program.

Multinucleation and large cell size are prominent features of osteoclasts, and were long suggested to be important for osteoclastic resorption (Bar-Shavit, 2007). It has been shown that in DC-STAMP-deficient mice osteoclast fusion is specifically disabled, resulting in formation of munonucleated cells that otherwise contain all the necessary resorptive machinery (Yagi et al., 2005). Importantly, these mononucleated osteoclast-like cells demonstrated significant reduction in their resorptive activity normalized to a single nucleus (Yagi et al., 2005). Direct comparison of osteoclasts containing different number of nuclei demonstrated that large osteoclasts have higher relative expression of many osteoclast markers including integrins α_v_ and β_3_, cathepsin K, and RANK compared to small osteoclasts (Trebec et al., 2007). However, whether it is the number of nuclei or the cytoplasmic size that is essential for osteoclastic resorption is not clear. Our data suggest that obtaining large size is an important objective of osteoclastogenesis, and that it can be attained either through monocyte fusion, or through fusion-independent cytoplasm growth, or through the combination of these processes. To understand why large cell size can increase osteoclast resorptive activity we should consider that the process of resorption occurs on the surface of the bone, therefore for an osteoclast with the radius of R, the area it can attach to and engage in resorption is proportional to *R*^2^. On the other hand, osteoclast function is based on the specific protein content within the volume of the cell, which with the increase in osteoclast size changes proportionally to *R*^3^. Therefore, a 10-fold increase in cell radius, from 10 μm normal for monocytic precursors to 100 μm common for osteoclasts, results in 10-fold increase in cell volume per unit area under resorption, providing 10 times more protein (assuming uniformity of protein content), such as proteases, for secretion, as well as ATP, necessary for ATPase function. Thus, we propose that it is overall cell size, rather than nucleation, that is important for osteoclast function, however whether osteoclasts target a predetermined cell size, or a maximal size that can be attained within a differentiation window remains to be resolved.

We have found that mTOR signaling is central for determining osteoclast size. Two distinct complexes can be formed by mTOR—mTORC1, which contains raptor as mTOR binding partner and regulates protein synthesis in part through phosphorylation of p70S6K and 4E-BP1, and mTORC2 with rictor as mTOR binding partner, which affects cytoskeletal organization and lipid metabolism (Sarbassov et al., 2005a; Foster and Toschi, 2009; Ma and Blenis, 2009; Laplante and Sabatini, 2013; Gaubitz et al., 2016). Previous studies demonstrated the important role of mTOR in osteoclast differentiation and survival (Glantschnig et al., 2003; Sugatani and Hruska, 2005; Hu et al., 2016; Dai et al., 2017). We have found that mTOR association with raptor and rictor was affected by the nutrient availability during osteoclast differentiation. In addition, Akt, which is also known to regulate osteoclast differentiation and survival (Sugatani and Hruska, 2005; Gingery et al., 2008; Kwak et al., 2008), was found to be a downstream target of mTOR. Previous studies have suggested that mTORC2 directly regulates AKT activity (Sarbassov et al., 2005b), our data suggest that such regulation may occur in our experimental conditions. Pharmacological inhibition of Akt resulted in strong decrease in fusion, which in energy-rich environment was compensated by increase in cytoplasmic growth. Of interest, we have previously demonstrated that inhibition of a mitogen activated protein kinase ERK1/2 during osteoclastogenesis also significantly decreased osteoclast nucleation while increasing cell area/nucleus (Tiedemann et al., 2009), suggesting that ERK and Akt pathways may be part of the same pathway that regulates osteoclast fusion. Based on these data, we hypothesize the following sequence of events: in control cells, mTOR is distributed between mTORC1 fraction that regulates protein synthesis (and thus transcriptional output) and mTORC2 fraction that through activation of Akt regulates cell fusion (and thus genome content). Energy-rich environment permits a large increase in transcriptional output, and thus mTORC1 fraction increases. In turn, since the protein synthesis requirements are met through upregulated translation, increase in genome content through cell fusion is not required, and thus mTORC2 fraction decreases. It has been previously demonstrated that all osteoclast nuclei are similarly engaged in transcription, and that transcriptional activity of osteoclast nuclei is strongly upregulated when osteoclasts engage in resorption (Boissy et al., 2002). Our data suggest that genome content and transcriptional output are closely regulated during osteoclast formation as well as osteoclast function.

Energy availability was found to significantly affect the execution and the outcome of the osteoclastogenesis process. We have previously demonstrated that metabolic sensor AMPK was significantly inhibited during osteoclastogenesis in the presence of pyruvate (Fong et al., 2013). AMPK is directly linked to energy metabolism, since it is activated by an increase in AMP reflecting cell inability to cope with energy demands. AMPK activation leads to energy conservation through inhibition of cell growth, lipogenesis and protein biosynthesis (Gwinn et al., 2008; Lage et al., 2008). mTOR is a target and a counterpart of AMPK signaling—activation of AMPK leads to mTOR inhibition, reducing energy used in protein synthesis. Cell growth and proliferation are known to be regulated by mTOR according to nutrient and energy status (Sarbassov et al., 2005a; Gwinn et al., 2008). In our studies, we have found that in the presence of pyruvate AMPK was inhibited, while mTORC1 complex was stimulated, leading to cell growth. Importantly, we have found that the presence of small amount of pyruvate, 1 mM, significantly decreased the effectiveness of mTOR/Akt inhibitors in reducing osteoclastogenesis. Taken together these data demonstrate that osteoclastogenesis is energy-dependent and bioenergetics-tailored process, and also suggest that bioenergetics microenvironment can significantly modify the effectiveness of inhibitors of involved pathways.

This study demonstrates that osteoclasts can attain large cell size through cell fusion thus increasing genome content, or through cell growth by increasing transcriptional output. Since osteoclasts of large size have been implicated in the pathological bone resorption, better understanding of the regulation of osteoclast size is important for development of selective inhibitors targeting osteoclasts actively engaged in pathological bone destruction, while preserving physiological levels of osteoclast activity.

## Author contributions

Study conception and design: DL, JF, JB, SK. Acquisition of data: KT, DL, JF, OH. Analysis and interpretation of data: KT, DL, JF, JB, SK. Drafting of manuscript: KT, DL, JF, SK. All authors contributed to the critical revision of manuscript and approved the final version to be published.

## Funding

This research was funded by the Canadian Institutes of Health Research (CIHR) operating grant number 77643 to SK and Natural Sciences and Engineering Research Council (NSERC) discovery grant to JB. DL was supported by the CIHR Training Program in Skeletal Health Research, the Canadian Arthritis Network and les Fonds de la Recherche en Santé du Québec. JF was supported by the CIHR Training Program in Skeletal Health Research and McGill University. OH was supported by the Merit Doctoral Research Scholarship from the Government of Quebec, by Lloyd Carr-Harris Fellowship and by McGill University.

### Conflict of interest statement

The authors declare that the research was conducted in the absence of any commercial or financial relationships that could be construed as a potential conflict of interest.

## Abbreviations

AMPK: AMP-activated protein kinase
4E-BP1: eukaryotic translation initiation factor 4E binding protein 1
mTOR: mechanistic target of rapamycin
DC-STAMP: Dendritic cell-specific transmembrane protein
OC: osteoclast
Py: pyruvate
RANKL: receptor activator of NF- κB ligand
Rapa: rapamycin
S6K: p70 S6 kinase.
